# Muriate of Ammonia in Nervous Cephalalgia

**Published:** 1860-09

**Authors:** 


### MURIATE OF AMMONIA IN NERVOUS CEPHALALGIA.

Professor Barallier, of Toulon, reports that within the last three years he had administered the substance in 259 cases of nervous cephalalgia, and with success in 202 of these. He gives forty-five grains combined with mint-water and syrup of orange-peel, divided into three doses, to be taken at intervals of half an hour, amendment commencing after the first dose, and the third frequently not requiring to taken. To prove effectual, however, the remedy should not be given at the very commencement of a paroxysm, but when it has acquired great intensity. This agent not only gives relief to the urgent pain of the paroxysms, but, after having been had recourse to on several occasions, diminishes the number and frequency of these. To be of use, it must not be indiscriminately used for every cephalalgia; and the result of the analysis of M. Barallier's experience leads to the following conclusions:

1. The muriate almost constantly dissipates paroxysms of idiopathic migraine, and of migraine consecutive to too abundant menstruation.

2. It is powerless in the hemicrania which is dependent upon irregularity or suppression of the menses.

3. It is tolerably successful in cranial pains dependent upon disorder of the stomach, and the accidental cephalalgia frequent in women and feeble persons under the influence of sudden changes of the atmosphere, prolonged intellectual labor, or moral emotion.

4. It operates beneficially in cephalalgias consecutive to repeated paroxysms of intermittent fever; those which are observed during the decline of severe fever, and in the course of the irritative period of typhus.—*Bull. de Therap and Dublin Med. Press.*

---

Rupture of Varicose Veins in the Vagina during Labor.—The *Berliner Zeitung* records the death of a young woman during labor with her first child, from rupture of the perineum, which involved a laceration of some varicose veins of the vagina. The patient died in ten minutes, from hemorrhage.

---

Drinking Fountains.—The first public drinking fountain established in London is used seven thousand times a day. The fountains are supplied by the water companies at the usual charge of sixpence per thousand gallons.

---

☞ M. Cloquet has been appointed President of the Academy of Medicine of Paris.

